# Phenotypic plasticity of bread wheat contributes to yield reliability under heat and drought stress

**DOI:** 10.1371/journal.pone.0312122

**Published:** 2025-03-10

**Authors:** Jatinder Singh Sangha, Weiwei Wang, Ron Knox, Yuefeng Ruan, Richard D. Cuthbert, Julio Isidro-Sánchez, Lin Li, Yong He, Ron DePauw, Asheesh Singh, Adrian Cutler, Hong Wang, Gopalan Selvaraj

**Affiliations:** 1 Swift Current Research and Development Centre, Agriculture and Agri-Food Canada, Swift Current, Saskatchewan, Canada; 2 Biotechnology-Plant Biology Department, Centre for Plant Biotechnology and Genomics UPM, Madrid, Spain; 3 Institute of Environment and Sustainable Development in Agriculture, Chinese Academy of Agricultural Sciences, Beijing, China; 4 Advancing Wheat Technologies, Calgary, Alberta, Canada; 5 Department of Agronomy, Iowa State University, Ames, Iowa, United States of America; 6 National Research Council of Canada, Saskatoon, Saskatoon, Canada; University of Idaho, UNITED STATES OF AMERICA

## Abstract

Yield reliability under diverse environments is important to address climate stress consequences in wheat production systems. Breeding for reliability under a changing climate remains a challenge in wheat. We assessed the performance of 18 hexaploid (*Triticum aestivum* L.) genotypes in three field environments at a location within the semi-arid Canadian Prairies over four years with a primary aim to establish knowledge of the phenotypic plasticity and yield reliability in the parental lines as it relates to heat and drought stress tolerance. We collected data on various physiological traits along with some agronomic and morphological attributes, uncovering significant variation across early seeded rainfed, early seeded irrigated, and late seeded rainfed (hot and dry) environments. Eight high yielding hexaploid genotypes ‘01S0263-28’, ‘AC Foremost’, AC Karma’, ‘Cutler’, ‘MN03358-4’, ‘Reeder’, ‘Stettler’, and ‘Superb’ showed higher grain Δ^13^C. Six of these genotypes ‘01S0263-28’, ‘AC Foremost’, ‘MN03358-4’, ‘Reeder’, ‘Stettler’, and ‘Superb’ showed higher water use efficiency under irrigated as well as hot and dry environment compared to the low yielding lines ‘Red Fife’,’8021-V2’ and ‘BW278’. Only four genotypes ‘01S0263-28’, ‘MN03358-4’, ‘Reeder’, and ‘Stettler’ were found with higher yield reliability index. The grain yield relationship with leaf rolling, glaucousness, and canopy temperature was found to be weak. The flag leaf stomatal numbers increased with water stress in high yielding lines which were otherwise low in stomatal numbers. Contrastingly, water stress significantly reduced the stomatal numbers in low yielding lines that were otherwise high in stomatal numbers. The results highlight the stomatal adaptability of different genotypes in response to drought. Taken together, these results provide baseline information that the genotypes with high grain Δ^13^C and WUE, and low stomata numbers are more yield reliable under variable field environments, and this information can guide the breeding of climate-resilient germplasm that expresses consistent and reliable grain yield production in the semi-arid Prairies.

## Introduction

Wheat is a staple food for nearly 35% of world’s population. It is grown widely in North America, contributing approximately 10% of annual global wheat production and more than a third of the world’s export supply [1]. The demand for bread wheat (*Triticum aestivum* L.) is growing due to changing food habits and increasing global population [2]. Feeding the world thus needs a consistent increase in wheat yield over the coming decades [3].

Improving grain yield in wheat is a challenge under current climate patterns requiring integrating new technologies and traits in breeding [4–6]. The current rate of genetic gain and varietal development effort for wheat are not sufficient to tackle the challenges of global wheat production under severe heat and drought stress [1,2,7]. There is great variability in wheat yield due to genotype by environment interaction, an important consideration in varietal development [8]. With further destruction and conversion of forest lands for agriculture crop use being a poor option, increasing wheat production must rely on exploiting physiological plasticity in plants and identifying and incorporating new sources of tolerance or resilience to weather extremes [9,10]. Screening and selection within diverse germplasm based on heritable physiological traits has been useful in developing wheat lines for regions with water scarcity [11–13]. Such germplasm is a source for improving yield with less variability in diverse environments, with better adaptability and resilience to heat and drought stress.

Hexaploid wheat, one of the important field crops on the Canadian Prairies, is commonly grown under semiarid conditions representing more than 90% of bread wheat production in Canada [14]. With a relatively small proportion of acreage under irrigation, most wheat acreage is totally dependent on snow melt that enters the soil and on subsequent rains for growth and development on the Prairies, therefore, climate change induced heat and drought stress limits wheat productivity in the region [15,16]. Attaining the full potential of wheat grain yield requires further improvements in breeding for stress tolerance, adaptability, and resilience [17,18]. Developing wheat varieties with climate change resilience, especially for heat and drought stress is therefore imperative to global food security [19].

Heat and drought events could reduce up to 50% of the expected grain production [18,20]. The impact is more intense if these conditions occur during the grain fill period [21]. Heat and drought are realized in western Canada with huge impact on crops. During the past 100 years, at least 40 droughts have occurred in Canada and those observed in the 1930s, 1980s, early 21st century, and recently in 2021 left a huge impact on the wheat sector [22,23]. Drought in 2021 was more intense than previous drought events and felt throughout the Prairies [23] pressing the need to develop heat and drought tolerant cultivars with less damage potential under such conditions. Many spring wheat cultivars in North America have been identified as sensitive to heat and drought with a reduction in yield of up to 7.5% for every 1°C increase in temperature [1]. One option for addressing this issue is to have cultivars that maintain grain yield reliability under diverse growing conditions, which limits downward yield fluctuations to address yield losses under unpredictable environments [24].

Selection of wheat genotypes in diverse environments using a combination of agronomic, morpho-physiological, and quality traits could help germplasm development for reliable yield performance under heat and drought. Various traits such as stomatal density, stomatal conductance, canopy temperature, and stable isotope signatures especially under water stress are representative of drought stress response in plants [12,25–27]. Some of these traits such as Δ^13^C are important indicators of photosynthetic activity, WUE, and high grain yield in wheat [26,27]. These specific physiological traits also relate well with molecular and biochemical attributes of the plant making them more relevant candidate traits for genetic association compared to the broader traits of drought and heat tolerance [28]. Inclusion of these physiological parameters with selectable and heritable features in breeding strategies is thus important for abiotic stress adaptation [26,29,30].

We studied a panel of diverse hexaploid wheat genotypes, including the founder line ‘Red Fife’ and a few popular wheat cultivars in Canada (Table 1). These wheat genotypes carry a diversity of traits that have been used in developing mapping populations and identifying trait marker associations [31]. Except for a few reports, not much is known regarding the variation in morpho-physiological traits in the majority of these genotypes [32–35]. Using a combination of measurable traits in this field study, we investigated drought stress resilience and yield reliability in wheat under the semi-arid climate of the Canadian Prairies. The objectives are to i) determine the grain yield performance of a diverse panel of wheat genotypes that can lead to establishing knowledge of the genetic basis of heat and drought stress tolerance in Canadian wheat; ii) characterize the phenotypic plasticity of the wheat panel for physiological traits that potentially associate with yield reliability under variable environmental conditions, and iii) improve our understanding of the morpho-physiological heat and drought stress traits that are best to apply to wheat breeding.

**Table 1:** The year of release, pedigree, origin of the seed, and other important trait details of the 18 hexaploid wheat genotypes used in the study^a^.

Genotype	Year of release	Pedigree	Origin	Important trait
01S0263-28 (SY-Soren)	2011	Norpro/Kelby	Syngenta Seeds, Inc.	Moderate resistance to leaf rust. Good FHB resistance.
8021-V2	1991	Kenya 321.BT.I.B.I/Peck	AAFC, Swift Current	Pre-harvest sprouting resistance. Moderately susceptible to leaf rust, moderately resistant to stem rust, and resistant to both common bunt and loose smut.
AC Barrie	1994	Neepawa/Columbus/ BW90	AAFC, Swift Current	Resistant to leaf rust, stem rust and common bunt.
AC Cadillac	1996	BW90 * 3/BW553	AAFC, Swift Current	Resistant to leaf rust, stem rust and common bunt, *Lr34, SrCad, Bt10*.
AC Domain	1993	BW83/ND585	CRC & AAFC Winnipeg	High levels of pre-harvest sprouting resistance.
AC Foremost	1995	HY320 * 5/BW553//HY320 * 6/ 7424-BW5B4	AAFC Lethbridge, Swift Current, Winnipeg	Better resistance to stem rust, leaf rust, common bunt and loose smut. Semidwarf strong straw.
AC Karma	1994	HY320 * 5/BW553//HY358 by HY358/7915-QX76B2 (7915-QX76B2 = NB320/NB402//RL4137)	AAFC Swift Current	Resistant to prevalent races of common bunt, loose smut, leaf rust, and stem rust. Good noodle quality, particularly for color.
BW278	1999	AC Domain * 2/Sumai 3	AAFC Winnipeg	Carries *Lr16* (leaf rust resistance gene), with oviposition deterrence to orange wheat blossom midge.
Carberry	2009	Alsen/Superb	AAFC Swift Current	Resistant to rust (*Lr34, Lr46*) and common bunt and moderate resistance to loose smut. Good resistance to FHB.
Lillian	2003	BW621 * 3/90B07-AU2B	CRC, AAFC Winnipeg & Swift Current	Resistant to rust and bunt and pre harvest sprouting. Solid-stemmed cultivar, wheat stem sawfly resistance, high grain protein content, *Gpc-B1/Lr36*.
MN03358-4	2009	MN98389/MN97518	University of Minnesota Agricultural Experiment Station	Moderate resistance to FHB. Has Sumai 3 source for Scab resistance.
Cutler	1991	a reselection of the line GP25l. It derives originally from a F2 population of the cross Ciano ‘S’/4/Sonora 64/Yaqui 50E5//Gaboto/3/Inia’S’ obtained from CIMMYT	University of Alberta	Early maturing. Poor disease resistance.
Red Fife	1842	‘Red Fife’ is a landrace	Galicia (Poland/Ukraine)	The unique milling and baking properties of Red Fife and its adaptation to the Canadian climate has made it the genetic parent to virtually all Canadian wheats grown in the prairies today.
RL4452	1977^b^	Glenlea * 6/KITT backcross	AAFC Winnipeg	*Lr34*. unregistered backcross derivative of the wheat cultivar ‘Glenlea’ with the dwarfing gene Rht-D1b.
Stettler	2008	Prodigy/Superb	AAFC Swift Current	High grain yield and high protein. Rust, bunt and loose smut resistance.
Superb	2001	single backcross of AC Domain to Grandin (Grandin * 2/AC Domain)	AAFC Winnipeg	Rust resistance and moderate resistance to common bunt. Good pre-harvest sprouting resistance. Genes: *Lr22, NO-Lr34, CSLV34A, NO-Lr46, PINA-D1A, PINB-D1B*.
Vesper	2011	Augusta/Hard White Alpha//3 * AC Barrie/5/BW150 * 2//TP/TM/3/2 * Superb/4/Grandin * 2/Caldwell/5/Superb	AAFC Winnipeg	Resistance to leaf rust and moderate resistance to stem rust, high yielding. Resistant to orange wheat blossom midge (*Sm1*)
Reeder	1999	IAS20 * 4/H567.71//STOA/3/ND 674	North Dakota	Rust resistance and good pre harvest sprouting resistance.

^a^Source: Crop Information Engine and Research Assistant (CIERA, 2023), Agriculture and Agri-Food Canada, Swift Current, SK.

^b^The year the cross was made.

## Materials and methods

### Plant material and field trials

We studied a panel of 18 hexaploid (*Triticum aestivum* L.) genotypes (Table 1) which are mostly bread wheats. These genotypes have been used in crossing within Agriculture and Agri-Food Canada’s (AAFC) breeding programs and are parents of genetic populations used to study traits such as disease resistance and abiotic stress tolerance. This panel also consists of three wheat lines from the USA (Reeder, MN03358-4 and 01S0263-28) and historical wheat lines that were used in decades past in Canadian breeding programs, including the founder line ‘Red Fife’. All the experiments were conducted at the research facilities of AAFC-Swift Current, SK. Therefore, no permits were required to use the facilities and complete this work. The research work was approved by the department.

In a multiyear field study from 2012 to 2014 and 2016, we seeded three replicated plots in three field environments on a Swinton loam soil (Orthic Brown Chernozem) near Swift Current, SK Canada using a randomized complete block design. The field environments were created as early rainfed (seeded early in May without irrigation, ES-RF), early irrigated (seeded in early may with irrigation, ES-IRR), and late rainfed (seeded in late May without irrigation, LS-RF). Each genotype was seeded in a plot of 3 m length and 1.2 m width having four planting rows, with 0.23 m between rows. The gap between plots was filled by seeding two rows of winter wheat. Each plot was sown at a rate of 1000 seeds per plot (adjusted for percentage of germination) with standard field practices.

### Phenotypic data collection

Data collected included measurements or documentation of traits associated with agronomy and morpho-physiology of the plants of each wheat line/cultivar to differentiate genotypic characteristics and responses under irrigated and rainfed environments.

#### Agronomic traits.

For agronomic data, we collected harvested grain yield (GY, kg ha^-1^), kernels per spike (KPS), test weight (TW), thousand kernel weight (TKW, g), days to anthesis (DTA), days to maturity (DTM), and plant height (PH, cm) and percent grain protein concentration (GPC, %). Harvesting was conducted using a Wintersteiger Elite plot combine (Wintersteiger AG, Salt Lake City, UT, USA) at 18% plant moisture on a wet weight basis. Grain samples were dried to about 12% moisture before weighing plot yield, expressed in kg ha^-1^. The number of KPS were counted using five spikes per genotype collected from the field environment. The TW was measured using methods described by the Canadian Grain Commission that uses volume of grain expressed in kilograms per hectolitre (kg/hL) (https://grainscanada.gc.ca/en/grain-quality/) and TKW was determined by weighing 1,000 seeds in grams. The number of DTA was recorded when 50% spikes showed anthers protruding in a plot whereas the number of DTM was recorded when >  75% of the spikes in a plot showed total loss of green color. The PH was measured using a meter stick, recording an average of three plants per plot. For grain quality, we measured GPC by scanning whole grain with a visual near infra-red spectrometer (FOSS NIRSystems 6500) at 13.5% moisture basis.

#### Morpho-physiological traits.

Under morpho-physiological traits, we collected canopy temperature (CT), carbon isotope discrimination (Δ^13^C), water use efficiency (WUE), glaucousness (GLA), leaf rolling (LR), and flag leaf stomatal density converted to stomatal numbers (SN) per flag leaf.

Canopy temperature of plants was measured using an infrared thermal imaging camera (FLIR T620) between 11:00 a.m. and 1:00 p.m., at late grain-filling, by positioning the infrared sensor at a consistent distance above the canopy and angled at 45° from the plot’s north side. We then used the temperature images produced by the image processing software (FLIR Research) to compute the average temperature for the entire plot canopy.

We used finely ground grain from the wheat genotypes harvested in 2012 and 2013 for carbon isotope analysis at the isotope facility of AAFC’s Research Centre in Lethbridge AB, utilizing the Finnigan Delta V isotope ratio mass spectrometer (Thermo Electron, Bremen, Germany). The stable isotope analyses used 0.3 mg of sample flour and reference material weighed into silver capsules. The process involved calculating the amount of ^13^C isotope in relation to the international Vienna-Pee Dee Belemnite (V-PDB) standard [36]. We calculated Δ^13^C as described in the handbook Physiological Breeding II [37] as follows:


$${\text{Δ}}^{13}\text{C}=\left[\left(\text{δa}-\text{δp}\right)\right]/\left[1+\left(\text{δp}/1000\right)\right]$$

Where δa is the stable carbon isotope composition of the atmosphere and δp refers to the stable carbon isotope composition of the plant sample.

Using soil moisture, precipitation, and grain yield data for the year 2012, we calculated the WUE for all wheat genotypes. We determined the soil water content up to a depth of 100 cm in different plots both at the onset of seeding and during crop harvest using a PR2 Soil Moisture Profile Probe (Delta T Devices, UK). Throughout the growing season, we conducted moisture readings at soil depths of 10, 20, 30, 40, 60, and 100 cm. For each measurement instance, we recorded three readings per plot. Using the grain yield data from 2012, we determined WUE ((kg ha^-1^mm^-1^) with the following equation:


$$\text{WUE= }\left(\text{Mean grain yield of wheat genotype/Total water consumed during crop growth period}\right)\text{*100}$$

We used methods described in the handbook Physiological Breeding II [37] to collect GLA notes in the early morning during the grain-filling period using the scale from 0 (none) to 10 (total cover of white waxy bloom on the leaves and stem). For LR, we rated plants during the grain-filling period in the early morning (before 10:00 a.m.), using a scale from 0 to 3. The extent of leaf rolling of the most recent fully expanded leaf was estimated as 0 =  no leaf rolling, 1 =  leaf loosely rolled from the tip (<33%), 2 =  leaf moderately rolled (34-66%), and 3 =  leaf tightly rolled (>67%).

We analyzed total number of stomata per flag leaf using stomatal density (mm^2^) from each wheat genotype grown in water stress (WS, drought) and non-stressed (WW, well-watered) conditions in a glasshouse experiment conducted from March 2023 to June 2023. Plants were grown in 1 L plastic pots filled with a mixture of field and peat soil. The greenhouse temperature was set at 15˚C for the initial 30 d after seeding and thereafter at 22˚C with 16 h of light and 18˚C with 8 h of dark. Once the flag leaf fully expanded, we took impressions from both the abaxial and adaxial surfaces using clear nail polish in the dry impression method described by Ferris et al. 2002 [38]. Using a stereo microscope attached to a digital camera (Optica, Italy), we captured images of the impressions at 10x magnification, ensuring a consistent field of view and manually recorded the number of stomata within 1 mm^2^ field of view (number of sample counts =  9). We determined the stomatal numbers per flag leaf area by multiplying the average stomatal density in mm^2^ by the average leaf area (LA), where LA =  [flag leaf length (mm) ×  flag leaf width (mm) ×  0.75].

### Data analysis

We analysed the data with Linear mixed model in R software (version 4.3.1) for variance components, with genotype effect considered as a fixed factor and environmental effect along with its interaction with genotype effect as random factors. Least square means were estimated in each experiment for each genotype for each trait using R software (version 4.3.1). Unless otherwise indicated, results were considered statistically significant if p < 0.05 or highly significant at p < 0.01. Figs in the manuscript were constructed with MS-Excel, JMP version 17.2 and SigmaPlot version 14.0 (Systat Software, San Jose, CA, USA). Using pairwise correlation analysis, we assessed the relationships between various traits within each environment and year, then visualized these correlations in a scatterplot matrix. Biplots of principal component analysis (JMP version 17.2) were performed to explain the variation influenced by different factors.

We computed broad sense heritability using the R package “inti”, following the equation provided below: https://cran.r-project.org/web/packages/inti/vignettes/heritability.html


$${\text{h}}^{2}={{\text{σ}}^{2}}_{\text{g}}/{\text{σ}}^{2}\text{p}$$

where *σ*^*2*^_*P*_ is the phenotypic variance component and *σ*^*2*^_*g*_ is the genotypic variance component.


$${\text{σ}}_{\text{g}}^{\text{2}}\text{=Σ}{\left(\text{g – x}\right)}^{\text{2}}{\text{/n}}_{\text{g}}$$

$${\text{σ}}_{\text{p}}^{\text{2}}=\;{\text{σ}}_{\text{g}}^{\text{2}}+\;{\text{σ}}_{\text{g·e}}^{\text{2}}{\text{/n}}_{\text{e}}+\;{\text{σ}}_{\text{ε}}^{\text{2}}{\text{/n}}_{\text{e·}}{\text{n}}_{\text{r}}$$

where n_g_ is the number of samples, n_e_ is the number of environments, n_r_ is the number of replications.

For estimating phenotypic coefficient of variance (PCV, %) and genotypic coefficient of variance (GCV,%) we used the formulas:


$$\text{PCV\%= }\left[\left({\text{sqrt σ}}_{\text{p}}^{\text{2}}\right)\text{/x}\right]\text{* 100 and}$$

$$\text{GCV\%= }\left[\left({\text{sqrt σ}}_{\text{g}}^{\text{2}}\right)\text{/x}\right]\text{* 100,}$$

where x is the sample mean.

We calculated yield reliability index (YRI) for grain yield in three field environments based on the method of Kataoka 1963 [39] as described in Doring et al. 2015 [24]:


$$\text{YRI}=\text{mGY}-\text{Z}\left(\text{P}\right)\text{x Sev}$$

where mGY is the mean grain yield, Sev is square root of the environmental variance, Z(P) is the percentile from the standard normal distribution for which the cumulative distribution function reaches the value P. Z(P) is 0.675 for P =  0.75; 0.840 for P =  0.80; 1.040 for P =  0.85; 1.280 for P =  0.90; and 1.645 for P =  0.95. For this study, we used Z(P) as 1.645 with the P value set as 0.95. A genotype with high YRI is considered more reliable for grain yield.

## Results

### Weather conditions during field trials

**Fig 1:**
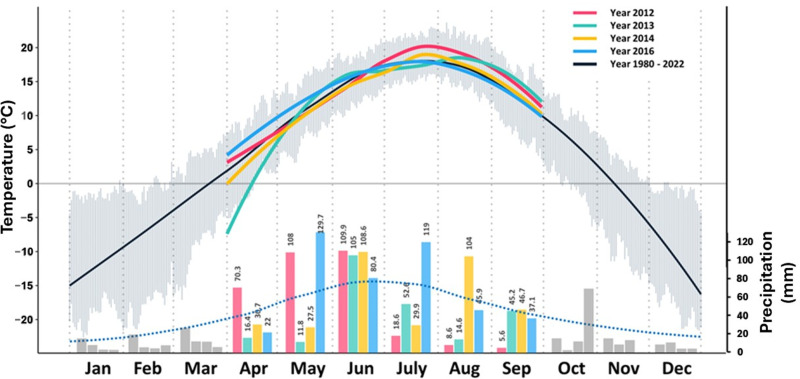
Precipitation and temperature for Swift Current, SK. Monthly average precipitation for the growing season of each year of the study is conveyed by colored bars with the scale in mm on the right-hand axis. The long-term average (1980-2022) precipitation is provided by the blue dotted line. The average temperature for the long term is provided by the solid black line, with the grey vertical streaks in the background indicating average daily extremes. Average daily temperature for each month for the years of study (2012-2014, 2016) is provided by solid-colored lines. The temperature scale is provided on the left-hand axis.

The long term historical (1980-2022), and trial years (2012, 2013, 2014, and 2016) precipitation and temperature data for different growing months at Swift Current are given in Fig 1. The rainfall in June for all four years was higher than the average of the last four decades. Late season drought occurred in 2012 and 2013 during the grain fill period of July and August with less precipitation than the long-term average. Less precipitation was received in July 2014 and August 2016 compared to the long-term average. A cooler growing season, favourable for vegetative growth, was experienced in 2013 with the highest temperature not exceeding 30^o^C.

### Phenotypic and genotypic variation in wheat traits

Data from various agronomic and morpho-physiological traits studied with hexaploid wheat lines showed considerable phenotypic and genotypic variation in the test lines. The mean squares for 13 of the 14 traits representing agronomic and morpho-physiological parameters studied in 18 hexaploid genotypes are presented in Table 2 whereas the estimates of σ^2^_g_, σ^2^_p_, PCV%, GCV%, and H^2^ for the data are given in Table 3. Genotype values for different agronomic and agronomic traits are given in Table 4.

**Table 2:** Mean squares and statistical significance of genotypes (G), environments (E) and interactions (GxE) for measured traits in hexaploid wheat.

Trait	G	E (Fields across all years)	GxE
**Agronomic traits**			
GY (kg/ha)	451094.5**	1896762.9**	530132.7**
KPS	65.3**	47.3**	24.1 **
TW (kg/hL)	101.0 **	266.5 **	104.8 **
TKW (g)	22.6 **	24.4 **	15.7 **
DTA (days)	16.8 **	341.9 **	12.2 **
DTM (days)	12.9 **	218.1 **	13.5 **
PH (cm)	331.5 **	61.5 **	30.8 **
GPC (%)	1.9 **	4.4 **	0.7 **
**Morpho-physiological traits**			
GLA	1.5 **	1.9 **	0.8 **
LR	0.5 **	1.1 **	0.5 **
Δ^13^C	1.0 **	6.1 **	0.4 **
CT (°C)	1.0 ns	67.5 **	0.8 ns
SN	19089411764.7**	3672000000 ns	28805882352.9**
WUE (kg ha^-1^mm^-1^)	7.89 **	12.62 **	3.88 **

*= Significant difference at p < 0.05; ** =  Significant difference at p < 0.01; ns = No significant difference; a = based on one year data. GY = mean grain yield, kg/ha; KPS = kernel per spike; TW = test weight, kg/hL; TKW = thousand kernel weight; DTA = days to anthesis; DTM = days to maturity; PH = plant height, cm; GPC = grain protein concentration, %; GLA = glaucousness; LR = leaf rolling; Δ^13^C = carbon isotope discrimination; CT = canopy temperature, °C; SN = stomatal numbers per flag leaf; WUE = water use efficiency, kg ha^-1^mm^-1^.

**Table 3:** Mean and range of measured agronomic and morpho-physiological traits, variance estimates, and broad-sense heritability (H^2^) in hexaploid wheat grown under three field environments.

Trait	Environment	Mean of three environments	Range (Min-Max)	σ^2^_g_	σ^2^_p_	GCV (%)	PCV (%)	H^2^
GY (kg/ha)	ES-RF	4000	3188-4633	109496	177354.1	8.3	10.5	0.61
	ES-IRR	4288	3504-4988	89980	143148	6.9	8.8	0.63
	LS-RF	3480	2818-3899	48849	75616	6.3	7.9	0.64
KPS	ES-RF	32	26-40	16.72	18.94	12.8	13.6	0.88
	ES-IRR	31	27-38	8.78	12.04	9.5	11.2	0.73
	LS-RF	29	25-36	11.16	13.92	11.5	12.8	0.8
TW (kg/hL)	ES-RF	310	299-323	33.43	43.73	1.8	2.1	0.76
	ES-IRR	310	297-322	40.7	54.95	2.0	2.4	0.74
	LS-RF	314	303-323	22.74	27.64	1.5	1.7	0.82
TKW (g)	ES-RF	33	25-36	5.54	6.82	7.1	7.9	0.81
	ES-IRR	35	27-39	5.55	7.31	6.7	7.7	0.76
	LS-RF	33	26-38	6.52	7.78	7.7	8.4	0.84
DTA (days)	ES-RF	66.4	65-71	1.49	2.468	1.8	2.4	0.60
	ES-IRR	68.8	66-73	2.26	3.1	2.2	2.6	0.73
	LS-RF	66.7	64-71	1.648	4.04	1.9	3.0	0.40
DTM (days)	ES-RF	99.7	96-103	2.158	3	1.5	1.7	0.72
	ES-IRR	105	100-105	2.57	3.28	1.5	1.7	0.78
	LS-RF	97	95-102	0.998	2.766	1.0	1.7	0.36
PH (cm)	ES-RF	95	76-113	123.02	124.94	11.7	11.7	0.98
	ES-IRR	95	74-117	138.64	140.61	12.4	12.5	0.98
	LS-RF	93	73-108	76.86	79.08	9.4	9.6	0.97
GPC (%)	ES-RF	12.9	11.3-14.3	0.448	0.51	5.2	5.5	0.88
	ES-IRR	13.5	11.5-15.3	0.73	0.81	6.3	6.6	0.90
	LS-RF	12.9	11.4-14.3	0.63	0.69	6.1	6.4	0.91
GLA^a^	ES-RF	1.3	0.2-2.3	0.21	0.29	34.1	40.0	0.72
	ES-IRR	0.8	0-1.7	0.135	0.227	46.5	60.3	0.59
	LS-RF	1.5	0.2-3.2	0.49	0.59	45.4	49.9	0.83
LR^a^	ES-RF	1.3	0.2-2.3	0.21	0.29	34.2	40.2	0.72
	ES-IRR	0.2	0-1.3	0.01	0.057	50.0	100	0.17
	LS-RF	0.9	0-2.3	0.236	0.36	54.6	67.4	0.65
Δ^13^C^a^	ES-RF	16.8	15.4-18.1	0.34	0.38	3.5	3.7	0.89
	ES-IRR	17.9	17.2-18.9	0.176	0.225	2.3	2.6	0.78
	LS-RF	15.1	14.2-16.4	0.17	0.21	2.7	3.0	0.81
CT	ES-RF	27.6	26.8-28.1	0.029	0.13	0.6	1.3	0.22
	ES-IRR	26.3	25.6-26.7	0.004	0.03	0.3	0.6	0.15
	LS-RF	25.9	25.4-26.9	0.03	0.18	0.6	1.6	0.16
WUE^b^	ES-RF	12.72	10.34-15.91	2.37	2.63	12.1	12.7	0.89
	ES-IRR	13.2	11.2-16.5	2.527053	2.70	12.0	12.4	0.93
	LS-RF	17.3	11.4-20.1	4.856702	5.14	12.7	13.1	0.94
SN^b^	Water stress	426159.6	348943.8-509999.6	1249515731	2121060679	8.3	10.8	0.59
	Well-watered	402724.5	296745.3-620936.8	7661264620	8242539287	21.7	22.5	0.93

GY = Grain yield (kg/ha)TKW = thousand kernel weight; TW = test weight; PH=plant height; DTA = days to anthesis; DTM = days to maturity; KPS = kernels per spike; SN = stomatal numbers per flag leaf; ES-RF = early seeded rainfed; ES-IRR = early seeded irrigated, and LS-RF=late seeded rainfed.

^a^Data based on analysis of two years.

^b^Data based on analysis of one year.

**Table 4:** Mean values of agronomic and morpho-physiological traits of hexaploid wheat under three field environments.

Environment	Genotype	GY	KPS	TW	TKW	DTA	DTM	PH	CT	Δ^13^C	WUE	GLA	LR	GPC	SN^a^
ES-RF	**01S0263-28**	4633	36	65	29	66	96	76	28.1	17.3	**15.7**	2.1	0.3	13	409727
	**8021-V2**	3406	29	63	36	67	102	105.7	27.5	15.8	12	1.9	1	12.8	385095
	**AC Barrie**	3935	30	65	34	66	100	104.6	27.3	16.5	13.2	1.6	1	13.6	348944
	**AC Cadillac**	4132	26	67	34	65	99	111.6	27.6	16.1	11.4	1.1	1.7	13.3	407885
	**AC Domain**	3697	30	64	32	64	99	101.3	27.4	16.9	11.9	1.1	1	13.8	476521
	**AC Foremost**	4207	34	62	34	66	100	78.3	28	17.4	15.9	2.3	1	12	433195
	**AC Karma**	4368	39	62	34	67	100	89.5	28.1	17.1	11.9	1.5	1.3	11.3	416201
	**BW278**	3188	31	65	26	67	101	106.7	27.5	16.8	10.3	0.7	2	13.5	430578
	**Carberry**	4185	28	65	34	65	99	88.1	27.7	16.3	12.8	1.2	1.3	13.3	352280
	**Cutler**	3841	33	62	32	65	96	82.8	27.8	18.1	13.2	1.9	2	12.5	429844
	**Lillian**	4113	26	64	34	68	100	103.1	27.5	16.8	11.8	1.1	1	14.3	429266
	**MN03358-4**	4612	38	65	30	66	100	86.7	28.1	16.7	13.7	0.2	2	13	473499
	**Red Fife**	3212	31	64	32	71	103	113.1	27.3	15.4	11.1	1.4	2	12.5	450940
	**Reeder**	4276	31	65	33	68	101	86.5	27.8	17	14.8	1.1	1	13.1	420798
	**RL4452**	4070	40	62	36	67	102	91	27.8	17	10.6	1.7	2	12.3	510000
	**Stettler**	4251	34	65	32	66	99	93.6	26.8	17	13.8	1.5	1.3	13.5	352836
	**Superb**	4151	28	63	36	66	99	90	27.3	17.2	13.4	1.4	1.3	12.5	481495
	**Vesper**	3725	29	65	34	66	99	99.6	27.2	16.8	11.7	0.5	1.7	13.2	461771
ES-IRR	**01S0263-28**	4488	37	65	30	67	104	74.6	26.4	18.3	15.1	1.3	1.3	13.7	381162
	**8021-V2**	3821	29	63	37	69	108	103.4	26.6	17.4	12	1.6	0	12.8	423225
	**AC Barrie**	4062	29	65	36	69	105	103.3	26.5	18.1	12.6	0.9	0	14.6	296745
	**AC Cadillac**	4368	28	66	37	68	104	111.9	26.7	17.5	11.5	0.5	0	13.9	321704
	**AC Domain**	3911	29	64	35	67	104	100.4	26.6	18.4	11.2	0.7	0	14.7	352647
	**AC Foremost**	4332	33	61	35	67	104	77	25.9	18.9	16.5	1.7	0.3	12.3	326702
	**AC Karma**	4988	37	63	36	70	105	89.2	26.3	18.6	15	0.5	0.2	11.6	383689
	**BW278**	3504	34	65	27	72	107	107.5	26.2	17.3	11.3	0.1	0.2	14.2	376998
	**Carberry**	4462	29	65	36	67	104	87	26.3	17.5	13.3	0.4	0.2	13.9	325767
	**Cutler**	4015	34	62	34	66	100	82.7	26	18.2	14.2	0.9	0.3	13.2	416084
	**Lillian**	4272	29	63	36	71	106	103.9	25.9	17.9	11.4	1.2	1	15.3	490104
	**MN03358-4**	4709	38	66	33	69	103	84.4	26.5	17.9	15.1	0.1	0.2	13.6	372887
	**Red Fife**	3863	31	65	34	73	108	116.7	26.2	17.3	11.8	0.6	0.3	13.1	620937
	**Reeder**	4649	29	65	35	68	106	88.5	25.8	17.7	13.8	0.7	0.2	13.7	426737
	**RL4452**	4447	35	62	39	71	107	90.6	25.6	18	11.7	1.1	0	12.8	618222
	**Stettler**	4774	35	65	34	69	105	94	26.1	18.3	14.4	1.1	0	14.1	370843
	**Superb**	4337	27	63	37	68	105	90.9	26.5	18.3	14.3	0.8	0.2	13.5	371011
	**Vesper**	4176	30	64	35	67	102	102.1	26.5	18.2	12.7	0	0.3	13.3	373578
LS-RF	**01S0263-28**	3619	31	66	31	66	101	75.3	26.2	15.4	18.9	1.5	0.3	13.5	–
	**8021-V2**	3211	29	64	34	68	99	101.1	25.7	14.6	16.9	2.7	0	12.2	–
	**AC Barrie**	3362	26	65	34	66	99	100.9	25.5	14.9	18.2	0.9	0.5	13.9	–
	**AC Cadillac**	3463	27	67	35	65	97	108.5	26	14.2	17	1	0.8	13.4	–
	**AC Domain**	3254	25	64	33	63	96	94.1	25.6	15	16.6	1.4	0.7	14.2	–
	**AC Foremost**	3557	36	64	35	69	97	80.3	26.6	15.6	19.8	3.2	1.2	11.9	–
	**AC Karma**	3899	36	64	33	71	99	92.4	26.9	15	16.6	1.4	0.5	11.4	–
	**BW278**	2818	29	66	27	67	97	97.8	26	15.2	14.8	1.1	1.2	13.3	–
	**Carberry**	3308	25	66	35	64	97	89.2	26.1	14.9	16.6	1.3	1.2	13.9	–
	**Cutler**	3462	28	64	37	64	95	84.1	26.5	15.7	14.4	2.5	2.3	12.4	–
	**Lillian**	3485	26	64	34	68	98	98.1	26.3	15.1	17.9	1.7	0.2	14.3	–
	**MN03358-4**	3884	33	66	31	67	97	85.9	26.4	14.8	20.1	0.3	1.3	13	–
	**Red Fife**	3127	29	66	31	68	99	106.4	25.6	14.5	15.6	1.8	0.2	12.2	–
	**Reeder**	3797	29	66	34	68	98	85.4	26.2	15	19.4	1	0.8	12.8	
	**RL4452**	3466	35	63	38	67	98	87.7	26.3	15.3	11.4	2.3	1	12.2	
	**Stettler**	3711	34	65	32	67	98	92.2	26.4	15.2	18.7	1.6	1.8	13.3	
	**Superb**	3648	26	65	38	64	96	90.8	25.4	15.3	19	1.6	1.3	12.7	
	**Vesper**	3577	28	65	34	64	95	98.9	25.5	15	19.9	0.2	0.7	12.6	

^a^Stomatal density (per mm^2^) recorded only under water stress and well watered conditions and expressed as stomatal numbers (SN) per flag leaf GY = mean grain yield, kg/ha; KPS = kernel per spike; TW = test weight, kg/hL; TKW = thousand kernel weight; DTA = days to anthesis; DTM = days to maturity; PH = plant height, cm; GPC = grain protein concentration, %; GLA = glaucousness; LR = leaf rolling; Δ13C = carbon isotope discrimination; CT = canopy temperature, °C; WUE, kg ha^-1^mm^-1^; ES-RF = early seeded rainfed; ES-IRR = early seeded irrigated, and LS-RF=late seeded rainfed.

#### Grain yield and other agronomic traits.

Analysis of the agronomic traits showed significant differences (p < 0.01) among the genotypes (G), and environments (field environments and years, E). The difference for G x E interaction was significant for all these traits (Table 2). Data on variance estimates showed that PCV estimates ranged from 1.7 for DTM to 13.6 for KPS and GCV estimates varied from 1.0 for DTM (LS-RFF) to 12.8 for KPS (LS-RF) in different field environments (Table 3). The highest σ^2^_p_ and σ^2^_g_ were found in GY and lowest were found in DTM and in DTA. Both σ^2^_p_ and PCV estimates were higher than the σ^2^_g_ and GCV of the agronomic traits, except for PH that showed the least difference in genotypic and phenotypic variance in all three environments. We observed low to high estimates of heritability for the agronomic traits. The H^2^ was the highest for PH in all field environments, whereas it was lowest for DTM (36%) followed by DTA (40%) under LS-RF environments.

With genotypes averaged over years, GY ranged from a minimum of 2818 kg/ha to a maximum of 4988 kg/ha under these environments (Table 3). The GY was highest under the ES-IRR field environment followed by ES-RF and lowest under LS-RF. Genotypic variation shows that the lowest GY was observed for ‘BW278’ in all three field environments, whereas the highest overall GY was recorded for ‘AC Karma’ under two field environments (ES-IRR and LS-RF). For the ES-RF field environment, GY was highest in the genotype ‘01S0263-28’ (Table 4). Similarly, smaller but significant variations were observed under these environments for other agronomic traits, KPS, TW, TKW, DTA, DTM, and PH. KPS was lowest in the LS-RF environment and TW was highest in LS-RF. Lower TKW was observed in both rainfed environments. Similarly, DTA was shorter in both rainfed environments, whereas DTM was shorter in the LS-RF environment and longer in the ES-IRR environment. The grain end use quality factor, GPC also increased significantly under the irrigated field environment, compared to the other two environments (ES-RF and LS-RF) ranging from a minimum of 11.3% to a maximum of 15.3% across field environments. Among genotypes, ‘AC Karma’ had the lowest GPC in all three field environments whereas ‘Lillian’ produced the highest GPC in all three environments (Table 4).

#### Morpho-physiological traits.

Analysis of the morpho-physiological traits in the wheat panel showed considerable variation among the genotypes and environments (Table 2).

#### Glaucousness and leaf rolling.

Both GLA and LR showed significant differences (p < 0.01) during field evaluation and showed genotypic and phenotypic variation (Table 2, Table 3). Both traits were significantly different for GxE interaction. The PCV and GCV estimates were higher for GLA and LR under all different field environments than other physiological traits (Table 3). The σ^2^_p_ and σ^2^_g_ values were low for both GLA and LR but σ^2^_p_ estimates were higher than the σ^2^_g_ and PCV estimates were higher than the GCV. The H^2^ was moderate to high for GLA. For LR, the trait was weakly expressed (17%) under ES-IRR conditions and among the lowest heritabilities for all the morpho-physiological traits studied in hexaploid wheat. The H^2^ for LR was moderate to moderately high for the other two environments.

GLA was expressed more in plants grown in LS-RF and ES-RF conditions compared to ES-IRR conditions (Table 3). However, the expression of GLA in wheat genotypes was not higher than three on a scale of zero to ten. During genotypic comparison, highest GLA was expressed in ‘AC Foremost’ in the LS-RF field environment, whereas the least expression was observed with ‘Vesper’ under irrigated conditions (Table 4). Similarly, genotypic variation was also observed for LR that was more evident in the LS-RF field environment with the highest LR recorded for Cutler (2.3). Although LS-RF produced the greatest range among cultivars for LR, ES-RF had the highest mean for LR. LR was least expressed in ES-IRR that affected parameters of variance estimates (Table 3).

#### Carbon isotope discrimination.

Genotypes differed significantly for grain Δ^13^C (p < 0.01), as did field environments (p < 0.01) and G x E interaction (p < 0.01) (Table 2). The variance components, σ^2^_p_, σ^2^_g_, PCV and GCV, were low for Δ^13^C in all three environments, although the σ^2^_p_ were higher than σ^2^_g_ and PCV estimates were higher than GCV. The heritability estimates were high in all three field environments.

When compared across field environments, grain Δ^13^C increased (p <  0.01) with decreasing water stress exhibited by lower mean values in the hotter later seeded rainfed environment (LS-RF) and early seeded rainfed (ES-RF) environments, compared to the cooler field environment with lower water stress (ES-IRR) (Table 3). When comparing genotypic variation under each environment, the lowest Δ^13^C values were observed with ‘Red Fife’ in the ES-RF (15.4) and ES-IRR (17.2) field environments, however, under the LS-RF environment ‘AC Cadillac’ had the lowest Δ^13^C (14.2) (Table 4). The genotype ‘Cutler’ was found with relatively high grain Δ^13^C in both rainfed environments (ES-RF = 18.1; LS-RF = 16.4), whereas the genotype ‘AC Foremost’ had the highest Δ^13^C (18.9) under irrigation (LS-IRR).

#### Canopy temperature.

Among all the morpho-physiological traits, we did not find significant genotypic differences (p > 0.05) in CT recorded at the grain fill stage in all three field environments, however, environment variation was significant at *p < *0.01 level (Table 2). The highest CT was observed in ES-RF (28.1˚C) and lowest in LS-RF (25.4˚C) (Table 3). Variance estimates showed that σ^2^_p_, σ^2^_g_, PCV and GCV were the lowest for CT in all three environments and not reliable for explaining H^2^ differences in CT estimates. The calculated H^2^ for CT was also low under all three environments and no H^2^ was calculated for ES-IRR. The analysis of genotypes for CT differences showed that ‘01S0263-28’, ‘AC Karma’ and ‘MN03358-4’ were highest in CT (28.1) under the ES-RF environment, whereas the genotype ‘Superb’ recorded the coolest CT (25.4) under LS_RF environment (Table 4).

#### Water use efficiency (WUE).

**Fig. 2:**
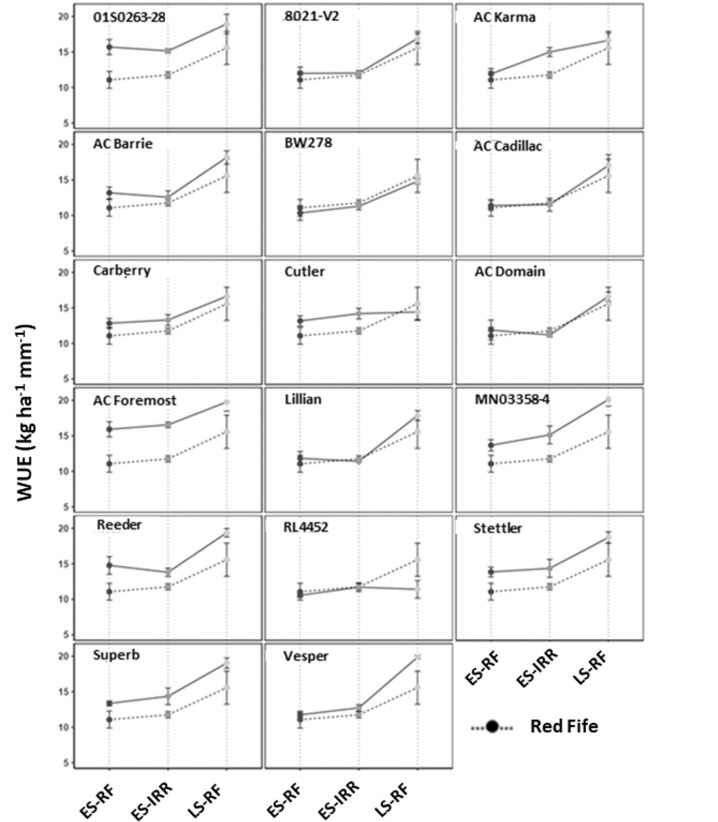
Comparison of water use efficiency (WUE) of the panel of hexaploid genotypes with Red Fife under three field environments. ES-RF = early seeded rainfed, ES-IRR = early seeded irrigated, and LS-RF=late seeded rainfed. The results are based on three replicates for each environment (Mean ±  SD).

Based on soil moisture data, crop season precipitation, and GY collected in 2012, we calculated WUE for all the hexaploid wheat genotypes grown in three field environments. The WUE of low yielding founder line ‘Red Fife’ was used as a reference for comparing the genotypes (Fig 2). Water use efficiency was higher in most high yielding modern hexaploid genotypes than the founder line, ‘Red Fife’ in all three field environments. Lines ‘01S0263-28’, ‘AC Foremost’, ‘MN03358-4’, ‘Reeder’, ‘Stettler’, and ‘Superb’ were consistently higher in WUE (p < 0.05) than ‘Red Fife’. However, WUE of ‘8021-V2’, ‘AC Barrie’, ‘BW278’, ‘AC Cadillac’, ‘AC Domain’, and ‘Lillian’ were found to be not significantly different (p < 0.05) from ‘Red Fife’ in any of the three field environments. Genotypes ‘Vesper’ and ‘RL4452’ showed significantly different WUE under the LS-RF environment, whereas ‘AC Karma’, ‘Carberry’, and ‘Cutler’ showed significantly higher WUE than ‘Red Fife’ under the ES-IRR environment.

#### Stomatal numbers on flag leaf.

**Fig 3:**
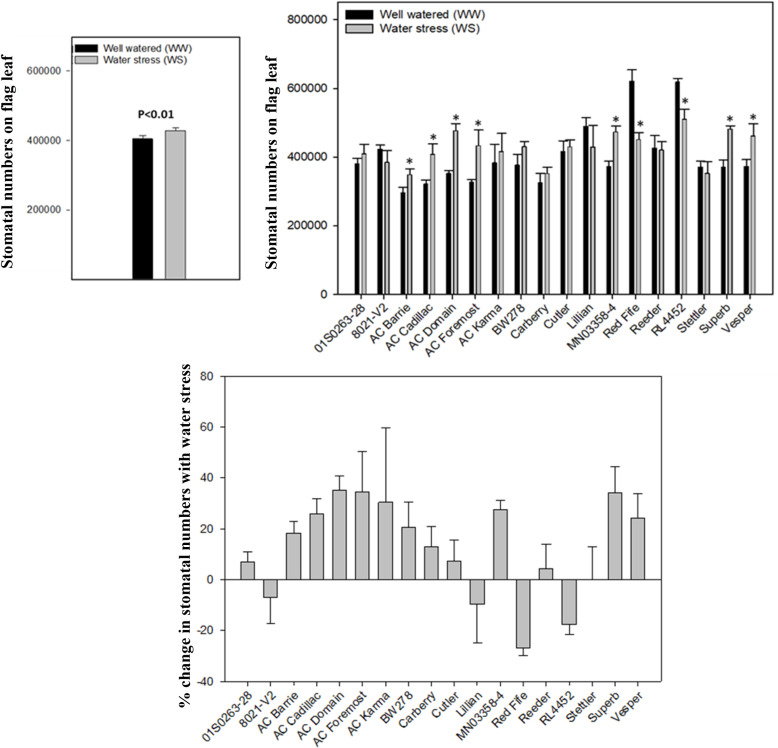
Variation in flag leaf stomata in hexaploid wheat grown under well-watered and water stress conditions. a) Average stomatal numbers of hexaploid flag leaf; b) genotypic variation in stomatal numbers combined for both surfaces of the flag leaf; c) percent change in stomatal numbers in well-watered and water stress conditions.

The stomatal numbers (SN) combined for both sides on the flag leaf varied significantly (p < 0.05) among genotypes grown under well-watered (WW) and water stress (WS) treatments. On average, under WW conditions, 402,724 stomata were observed on the entire flag leaf, whereas this number increased to 426,159 under drought stress (p < 0.05) (Fig 3a). Generally, the stomatal numbers consistently increased in high yielding lines, whereas it was found to decrease in low yielding lines, ‘Red Fife’, ‘RL4452’, ‘8021-V2’, and ‘Lillian’ (Fig 3b). Genotypic variation of stomatal numbers showed that the lower SN under WW conditions was found with ‘AC Barrie’ (296,745) and the highest number recorded was for ‘Red Fife’ (620,937). In the case of WS, the lowest stomata number was recorded for ‘AC Barrie’ (348,944) and highest number was found with ‘RL4452’ (509,999). When compared to other genotypes, eight genotypes showed non-significant change in stomatal numbers with water stress on both sides of the flag leaf.

‘Stettler’ showed the least change in SN with change in water regime (WW = 370,832 to WS = 352,836). The % change (decrease) in SN was higher in ‘Red Fife’, followed by ‘RL4452’, whereas the % change was lower in ‘Lillian’ and ‘8021-V2’ (Fig 3c). A similar trend for the change in SN was observed on both the adaxial and abaxial leaf surfaces (S1 Fig). On average, the adaxial stomata increased from 229,464 under the well watered condition to 242,095 with water stress, whereas abaxial stomata increased from 173,260 to 184,065.

As with stomatal numbers, the flag leaf area of wheat genotypes varied with water availability (S2 Fig). The average leaf area increased from 29.9 cm^2^ under WS conditions to 30.4 cm^2^ with WW. Under WW conditions, the greatest flag leaf area was observed in ‘RL 4452’ (42.20 cm^2^), whereas smallest leaf area was recorded with ‘AC Domain’ (23.3 cm^2^). When water stress was applied, the greatest leaf area was observed for the genotype RL4452 (33.9 cm^2^) and smallest for Carberry (25.9 cm^2^).

### Yield reliability index

Wheat genotypes with higher reliability index tend to be those with a higher grain yield and with low to moderate variance (S^2^) for the yield estimates across different environments. Four high yield hexaploid wheat genotypes such as ‘Reeder’, ‘Stettler’, ‘01S0263-28’, and ‘MN03358-4’ with low to moderate mean variance were found with comparatively high reliability index than other genotypes, with a YRI ranging from 2833 to 3147 (Table 5). The founder genotype ‘Red Fife’ (YRI = 2001) followed by ‘RL4452’ (YRI = 2106) were found to be among the least reliable hexaploid genotypes for grain yield among environments during this study. One line ‘BW278’ with low variance and low yield did not show high YRI. Another line, ‘AC Foremost’ is an example of a relatively modern high yielding genotype with a low reliability index due to a very high yield variance. Similarly, AC Karma showed comparatively high variance than other genotypes.

**Table 5:** Yield reliability index for hexaploid wheat grown for four years (2012-2014, 2016) in three field environments.

Genotype	Mean GY (kg ha^-1^)	Variance (S^2^)	Yield Reliability Index (p = 0.95)
Reeder	4240	441541	3147
MN03358-4	4401	649067	3076
01S0263-28	4247	632254	2939
Stettler	4245	737118	2833
AC Barrie	3786	399071	2747
Carberry	3985	648231	2660
Vesper	3826	540572	2617
Superb	4045	781815	2591
Lillian	3956	695412	2585
AC Cadillac	3988	841157	2479
AC Karma	4418	1425480	2455
AC Domain	3621	546202	2405
8021-V2	3479	441129	2386
Cutler	3772	891954	2219
BW278	3170	386806	2147
RL4452	3994	1318000	2106
Red Fife	3401	724424	2001
AC Foremost	4032	1811417	1818

### Relationship between various traits

**Fig 4:**
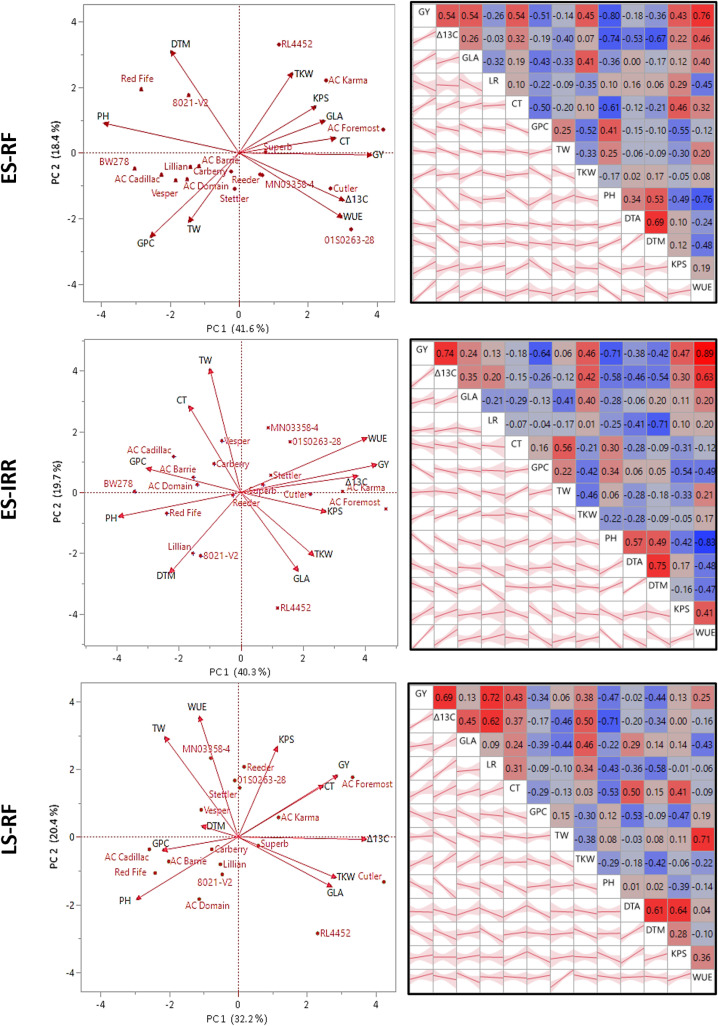
Principal Component Analysis biplot and color maps for pairwise correlations of measured traits (GY = grain yield kg/ha, Δ^13^C = carbon isotope discrimination, GLA = glaucousness, LR = Leaf rolling, CT = canopy temperature, GPC = grain protein concentration, TW = test weight, TKW = thousand kernel weight, PH = plant height, DTA = days to anthesis, DTM = days to maturity, KPS = kernel per spike, WUE = water use efficiency) in a hexaploid wheat panel grown under three field environments (ES-RF = early seeded rainfed, ES-IRR = early seeded irrigated, and LS-RF = late seeded rainfed).

Pairwise correlation analysis shows that many traits exhibited moderate to high correlations with grain yield of hexaploid wheat lines grown in three field environments. Among all genotypes grown in the ES-RF environment, a positive correlation of GY was observed with Δ^13^C, GLA, CT, TKW, KPS and WUE, whereas a negative correlation was observed with PH, GPC, DTM, and LR (Fig 4). The correlations of GY were very low (<-0.2) with DTA and TW. Under ES-IRR conditions, a similar trend was observed, except for a low negative correlation of GY with CT, TW, and LR. Under the LS-RF environment, the GY was found to have a high positive correlation with Δ^13^C and LR and a moderate to low positive correlation with CT, TKW, and WUE. GY was negatively correlated with GPC, PH, and DTM. The correlations of GY were very low with GLA, TW, DTA, and KPS. The Principal Component Analysis biplot of the hexaploid wheat traits shows that the first two principal components of the biplot reflected about 60% of the variance under ES-RF, 60% of the variance under ES-IRR, and 53% of the variance under LS-RF (Fig 4).

## Discussion

This study helped to determine the variability in grain yield performance to establish knowledge of the genetic basis of heat and drought stress tolerance in Canadian wheat, and characterize the phenotypic plasticity of wheat to apply the morpho-physiological traits in breeding for yield reliability under variable environmental conditions. From the field evaluation of a panel of 18 diverse hexaploid wheat genotypes including some newer and older cultivars, and advanced breeding lines, we found extensive evidence of phenotypic plasticity in different wheat lines. This phenotypic plasticity was evident from the variation observed in some agronomic and morpho-physiological traits in wheat lines grown under different environments. Such variability in germplasm is essential for genetic enrichment in yield related traits in cultivars for diverse field environments as it provides understanding of the traits required for the selection of climate resilient genotypes [6,19]. This information on phenotypic and genetic variability of traits is potentially useful for improving the yield reliability of Canadian wheat. Our study is specifically relevant to the rainfed Prairies that supports over 90% of the wheat production in Canada, which has experienced frequent drought events in recent years [23].

### Phenotypic and genetic variability in wheat

#### Grain yield and related traits.

The difference of 40% (2,170 kgs/ha) in harvested grain within 18 wheat lines grown in three field environments indicates the presence of diverse genetics among bread wheat lines for grain yield. In fact, all the agronomic traits except CT studied during four years presented significant variability among genotypes in different environments. The variance components, PCV and GCV that ranged from low (<10%) to high ( > 20%) estimates for the grain yield and other agronomic traits confirms that moderate phenotypic and/or genotypic variability exist in this wheat panel. Variation in agronomic traits is a key for cultivar improvement. In wheat, phenotypic and genetic variation for agronomic traits have been shown with a wide range of diversity [40,41]. Whereas the lines with high genetic variability in traits are considered more relevant for use in breeding, those with low genetic variability are still important in phenotypic plasticity [42]. These lines with low genetic variation for the traits are therefore useful to make contrasts for genetic assessment of various plant traits.

The PCV estimates for the agronomic traits were slightly higher than GCV indicating that there was environmental influence on these traits, as evident from the statistically significant GxE interaction. Since the difference between PCV and GCV was small, the magnitude of environmental influence was not high and most of the variation was due to existing genetic variation [43]. However, our study was already a multiyear environmental assessment and the results of the variability were based on consideration of GxE interaction in the model. Therefore, the results provide great potential for genetic assessment of yield related traits under tested field environments. Since response to selection for a quantitative trait is directly proportional to the function of its heritability and its genetic variance [44], the variance components observed in this study for the test material will enable wheat breeders to apply selections based on available genetic differences in the test lines. Low estimates of broad sense heritability indicate that genetic variance is low compared to the phenotypic variance. We found moderate to higher heritability estimates [45] in our results from 12 site years for the majority of the agronomic traits, which suggests that obtaining genetic gain for most of these traits through selection in different environments is possible [46].

### Morpho-physiological traits

Our study shows a wide range in the estimates of variance components for the morpho-physiological traits when compared to agronomic traits that we studied, which provides the baseline for the presence of substantial genetic variability in the wheat panel for morpho-physiological evaluation [43,44]. The estimates for PCV and GCV were higher for GLA and LR, but the heritabilities were lower, especially under the ES-IRR environment. The difference between PCV and GCV for GLA and LR were also higher suggesting that these two traits were highly influenced by the environments [44]. In contrast, the PCV and GCV estimates for other morpho-physiological traits were low and the difference between the two variance components was small suggesting lesser impact of GxE interaction on these traits, especially in Δ^13^C, WUE, and leaf SN.

The high broad sense heritability estimates for various morpho-physiological traits in the wheat panel that we analysed in this study also suggest a low genotype-by-environment interaction for many of the morpho-physiological traits, which is also in agreement with reports from others [45]. Since a high heritability for the plant traits is an indication of lower environmental influence, the selection based on phenotypic expression of individual accessions for these traits may be easy due to relatively small contribution of the environment to the expression of the phenotype where most of the variation is rather genetically influenced [46]. The high heritability estimates of the morpho-physiological traits in different environments including under the hot and dry environments we tested suggest that any progress made in selection of these traits in wheat can be of high breeding value for genetic advancement of water stress tolerance [47], particularly for germplasm development for yield improvement at the location of our study. These results improve our understanding of physiological breeding that will further improve grain yield reliability in wheat.

### Yield reliability in different environments

Yield reliability index helps to predict the minimum average yield for a genotype grown in diverse environments that a grower can expect from his crop [24,39,48]. In fact, reliability in yield is highly relevant to address yield fluctuations and losses under unpredictable environments [24]. The genotypes ‘Reeder’, ‘Stettler’, ‘01S0263-28’, and ‘MN03358-4’ were found with high YRI based on low to moderate variance estimates and high grain yield in different field environments across the years. The genotypes with high YRI will be useful for those environments with yield fluctuations [24]. While the YRI for the line ‘Red Fife’ was one of the lowest, another line ‘AC Foremost’ was less reliable for grain yield than ‘Red Fife’. The founder line ‘Red Fife’ is a tall cultivar, that was susceptible to severe drought, resulting in poor yield performance in dry environments [33]. There is some limited production of ‘Red Fife as a heritage cultivar, but not for large scale commercial growing. Interestingly, a number of modern hexaploid wheat cultivars and newer genotypes descended from ‘Red Fife’ were found with low mean variance for grain yield along with above-mean grain yield, that is to say have improved yield reliability. Consequently, this demonstration of improved reliability for grain yield through breeding along with the results of this study suggest further possibilities to increase genetic gain for climate resilience and yield reliability under water stress environments with physiological trait based selections as reported by others [18,49].

### Potential traits for drought stress selections

One of the morpho-physiological traits we focused on was Δ^13^C because it showed mostly a positive relationship with grain yield and genotypic variation in wheat for this parameter is also well documented [5,25,27]. It is reported that the environmental factors such as the availability of water during the crop growing period and the intensity of drought can influence the relationship of Δ^13^C with grain yield [25,50,51]. However, our results showed a consistent positive relationship of Δ^13^C with grain yield, along with the genotype variation and a high heritability of this trait. Wheat growing areas in the Prairies, including the location of the current study, often receive sufficient moisture and ground recharge from winter snow until anthesis, with subsequent dry spells affecting crop growth and development. The relationship between grain yield and grain Δ^13^C did not change between early and late sowing, the latter of which creates hot and dry conditions for the crop due to a shortage of available moisture during grain filling. It seems that this phenomenon showing a positive GY-Δ^13^C relationship could be common in the Prairies although the intensity could vary in different years. Such as in growing year 2012, which is recognized a drought year [23], we found a strong GY-Δ^13^C relationship compared to the less dry year 2013. These observations indicate the potential of Δ^13^C to be used in selections for drought stress tolerance and yield related traits. Measuring Δ^13^C on a small sample of grain from an F_2_ plant could benefit breeding hexaploid for yield improvement.

Crop plants that show improved water relations, such as water uptake, and optimized transpiration, including higher water use efficiency are desirable for regions that experience regular water stress [49]. Therefore, we focused on WUE as another trait in assessing wheat lines and found that WUE has been increased in many cultivars in parallel to the improvements in grain yield traits since the introduction of ‘Red Fife’ in the early 20^th^ century. The high yield modern hexaploid genotypes such as ‘Reeder’, ‘Stettler’, ‘01S0263-28’, and ‘MN03358-4’ displayed higher WUE compared to older low yielding genotypes ‘Red Fife’ and ‘RL4452’. WUE is a selection target for drought resilience in wheat. Some reports did reveal that drought tolerant crops with higher WUE could show lower grain yield [52]. Our findings are showing improvement in grain yield in lines with higher WUE and suggest that selecting wheat varieties with both high grain yield and the WUE is a possibility when developing drought tolerant wheat germplasm [51]. Many of the hexaploid lines such as ‘Reeder’, ‘AC Karma’, ‘Stettler’, ‘MN03358-4’, and ‘01S0263-28’ were selected and developed for rainfed semi-arid regions in the Prairies, which is likely the case for high WUE and grain yield than other genotypes. In fact, some of the high yielding genotypes in this study have performed well in the farmers fields over the years [53].

Stomata are important components in plant water relations and CO_2_ uptake for carboxylation in the chloroplast. A change in stomata density or the total number of stomata per leaf area with water stress can impact WUE and Δ^13^C content in the plant’s tissue thus altering yield [37,54,55]. We also observed variation in stomatal numbers among wheat genotypes grown under well-watered (no-stress) and water stressed conditions to find further evidence of phenotypic plasticity in hexaploid wheat. With the exception of four genotypes, our finding of increased stomatal numbers under water stress, is in agreement with the findings of other studies [reviewed in 56]. The decline in stomatal numbers of four genotypes, ‘8021-V2’, ‘Lillian’, ‘RL4452’, and ‘Red Fife’ after water stress is interesting, as this contrasting change in stomatal numbers per unit leaf area could have a relation with plants’ susceptibility response to drought. Reducing the stomatal numbers is considered important for improving WUE in plants to cope with water stress [54,55]. Perhaps the inherently reduced stomatal numbers and induced stomatal numbers with water stress in a genotype is unrelated and leads to different mechanisms of water stress response in plants. Thus, the reduction in stomatal density and numbers per flag leaf with water stress in low yielding lines ‘Red Fife’ and ‘RL4452’ may explain the plant’s attempt to adapt to a stressful environment, but with limits in the plasticity with other WUE traits. Consequently, the genotypes such as ‘Red Fife’ and ‘RL4452’ were more prone to water loss than other genotypes despite showing adaptive tendencies. The physiological mechanisms for this variation in stomatal number in hexaploid wheat is still not fully clear and needs further understanding.

Two lines, ‘Reeder’ and ‘Stettler’, that are found to be highly yield reliable, did not show a noticeable change in stomata numbers under both water regimes, whereas the others showed smaller change. In a recent published work [35], the genotype ‘Stettler’ showed lower stomatal conductance than another genotype ‘Superb’ under moisture stress (~60% of field capacity), which indicates the role of stomatal control to cope with water stress [35]. Additional information will be useful to understand the function of stomatal traits in relation with WUE in tested genotypes. Both ‘Reeder’ and ‘Stettler’ are found to be highly yield reliable and worth consideration as parents in crossing for incorporation of heat and drought tolerance in wheat for the Prairies. Other stomatal traits such as size and timing of opening and closing [5,55] may explain their role in regulating water stress mechanisms in hexaploid wheat.

### Relationship of GY with different traits

The relationship between Δ^13^C and WUE is known to be complex. While some reports describe a lower Δ^13^C with higher WUE in plants, others demonstrate a positive increase in Δ^13^C with increasing WUE [5,50,57,58]. This association is influenced by the factors such as availability of water and nutrients, soil properties, and species [59]. The association of GY with Δ^13^C and WUE was strong in our study, although the GY was poorly correlated with stomatal numbers. Improvements in crop genetics through physiological breeding with selective parameters such as Δ^13^C and WUE is realistic based on the development of wheat cultivars ‘Drysdale’ and ‘Rees’ for drought prone environments in Australia [11]. Our results corroborate those reports providing a further line of evidence that physiological traits such as Δ^13^C and WUE could be reliable physiological markers for germplasm screening. The performance of conventionally bred cultivars such as ‘Reeder’, with increased WUE and corresponding Δ^13^C suggests the possibility of selecting these traits for improving yield reliability in hot and dry rainfed agri-zones such as Canadian Prairies. However, WUE is extremely cumbersome to measure in a breeding situation, whereas Δ^13^C is technically feasible, where laboratory facilities are available to process samples from breeding programs.

Interestingly, one of the important physiological traits, CT was not well correlated with GY in different field environments within our study. This is in contrast to many other reports that showed CT as a good proxy for GY prediction [12,30,54]. Many factors such as plant density, height, and growth stage are known to introduce confounding results to CT that would not accurately predict GY or other associated parameters [54,60,61]. Since the wheat genotypes in the panel have divergent phenology and morphological features, we couldn’t identify the confounding factors influencing the CT-GY relationship [62]. We also conducted an extended experiment in 2023, to measure CT of the genotypes at different times of the day (7:00 a.m., 10:00 a.m., 1:00 p.m., 4:00 p.m., 7:00 p.m.), but no factors were identified for the inconsistent behaviour of CT results. Nevertheless, lower CT was observed for high yielding genotypes such as ‘AC Foremost’ compared to other genotypes with low yield such as ‘Vesper’ and ‘Red Fife’. These observations imply that to obtain accurate CT measurements, timing of observations relative to phenological stage and perhaps different and more reliable tools are required for in a hot and dry (rainfed) environment.

## Conclusion

Heat and drought are major limiting factors affecting crop productivity. Projected climate change and the possibility of continuous drought events in wheat growing regions will require that more and diverse wheat germplasm be evaluated to identify traits better adapted for improved water relations or other water saving mechanisms for reliable grain yield in hot dry regions, such as the Prairies. Development of germplasm with abiotic stress tolerance requires plasticity of traits that plants can utilize to adapt to changing weather conditions, without having significant yield penalty. Four yield reliable genotypes (Reeder, MN03358-4, 01S0263-28, and Stettler) found in this study are worth further investigation, due to showing less variation in yield in different field environments. In addition, wheat yield reliability could be improved through focusing on specific traits such as WUE and Δ^13^C and stomatal numbers per plant. Additional studies could help to better understand these findings. In fact, higher heritability of these traits suggests that measuring these physiological traits in an early generation of breeding would be feasible and a better means of enriching breeding populations for yield reliability before multi-environment yield trials are applied to the populations. For example, Δ^13^C can be determined on a single plant basis from grain of plants selected from large early generation breeding populations. Furthermore, genetic populations already developed from crosses of the genotypes evaluated in the present study can be used for genetic studies to find genes or QTL associated with yield reliability and traits such as Δ^13^C. Additional studies are underway to confirm the use of morpho-physiological traits, including Δ^13^C, WUE, and low stomatal numbers in selection of early generation wheat lines in diverse field environments through a physiological breeding approach.

## Supporting information

S1 Fig Stomatal numbers on adaxial and abaxial sides of the flag leaves.
Total number of stomata on flag leaf of hexaploid wheat grown in well-watered (WW) and water stress (WS) conditions in a glasshouse experiment. Data based on nine images taken on three independent flag leaves (Mean ± SE).
(PDF)

S2 Fig Flag leaf area of hexaploid wheat.
Mean flag leaf area of hexaploid wheat genotypes grown in well-watered (WW, blue solid line) and water stress (WS, red solid line) conditions in a glasshouse experiment. Data based on three randomly selected flag leaves for measurements of leaf area (Mean ± SE).
(PDF)
