# Supplementary material for: Phenotypic plasticity of bread wheat contributes to yield reliability under heat and drought stress
Source: PLoS One. 2025 Mar 10;20(3):e0312122. doi: 10.1371/journal.pone.0312122 (PMC11892852; doi:10.1371/journal.pone.0312122)
Supplement: S1 Fig — Total number of stomata on flag leaf of hexaploid wheat grown in well-watered (WW) and water stress (WS) conditions in a glasshouse experiment. Data based on nine images taken on three independent flag leaves (Mean ± SE). (PDF) [file pone.0312122.s001.pdf]

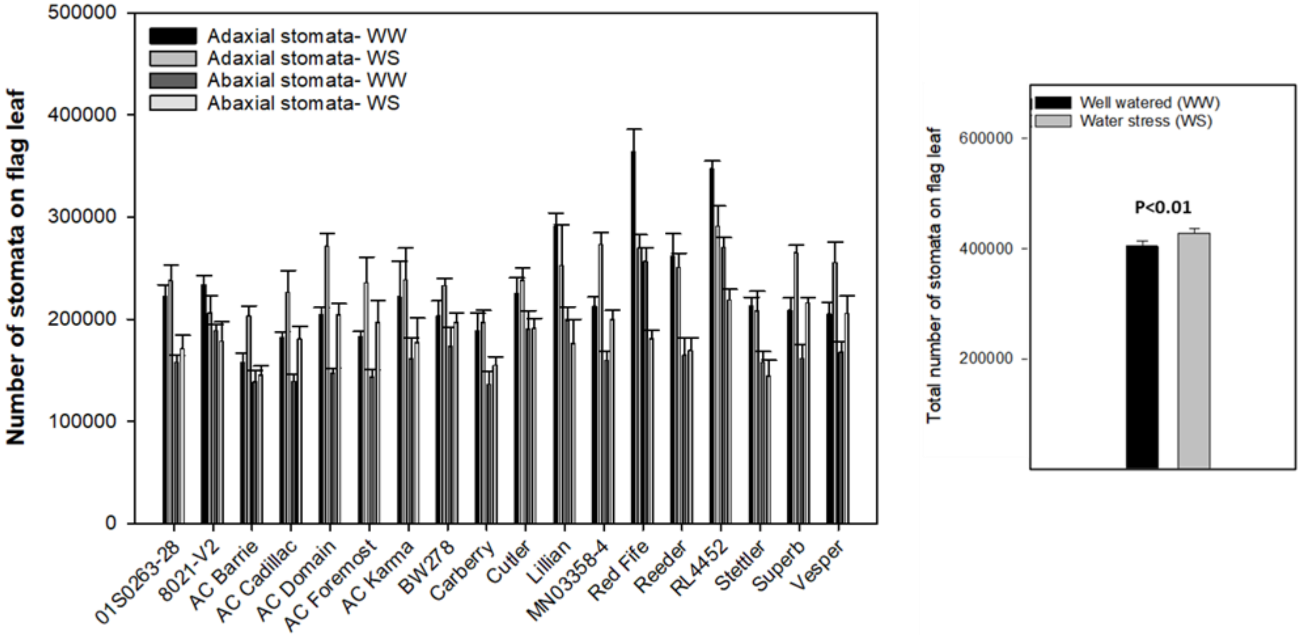

S1 Fig. **Stomatal numbers on adaxial and abaxial sides of the flag leaves.** Total number of stomata on flag leaf of hexaploid wheat grown in well-watered (WW) and water stress (WS) conditions in a glasshouse experiment. Data based on nine images taken on three independent flag leaves (Mean ±SE).
