# Supplementary material for: Phenotypic plasticity of bread wheat contributes to yield reliability under heat and drought stress
Source: PLoS One. 2025 Mar 10;20(3):e0312122. doi: 10.1371/journal.pone.0312122 (PMC11892852; doi:10.1371/journal.pone.0312122)
Supplement: S2 Fig — Mean flag leaf area of hexaploid wheat genotypes grown in well-watered (WW, blue solid line) and water stress (WS, red solid line) conditions in a glasshouse experiment. Data based on three randomly selected flag leaves for measurements of leaf area (Mean ± SE). (PDF) [file pone.0312122.s002.pdf]

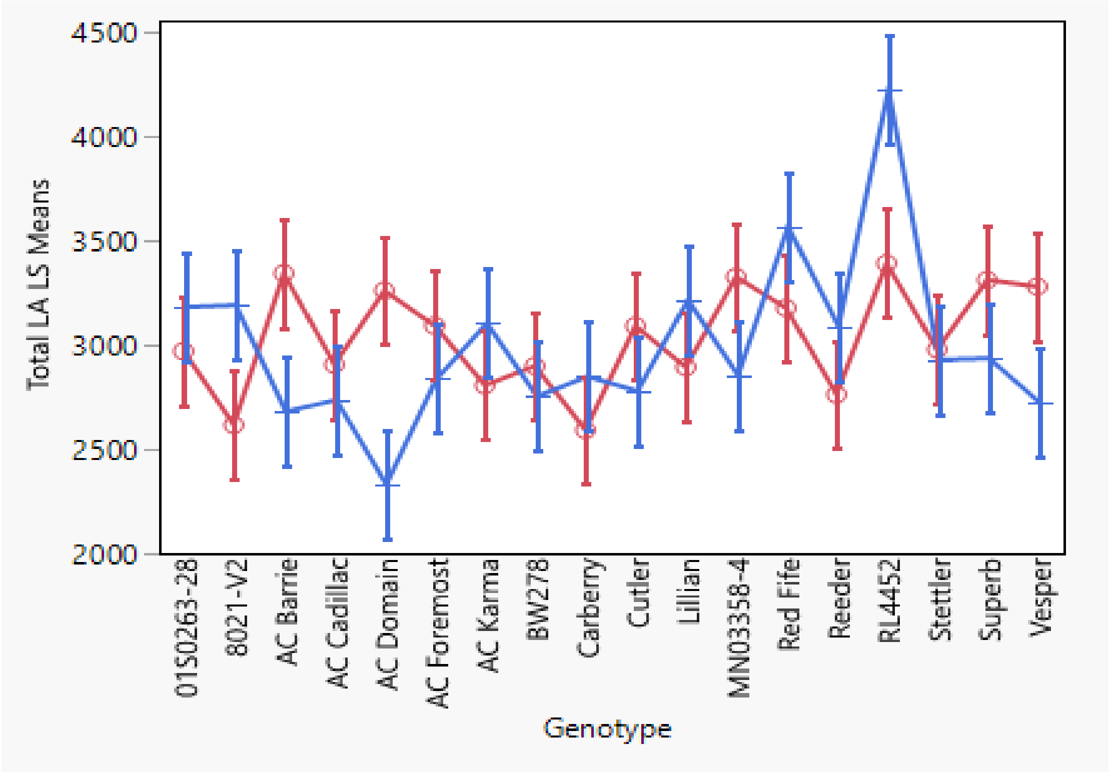


**S2 Fig**. **Flag leaf area of hexaploid wheat.** Mean flag leaf area of hexaploid wheat genotypes grown in well-watered (WW, blue solid line) and water stress (WS, red solid line) conditions in a glasshouse experiment. Data based on three randomly selected flag leaves for measurements of leaf area (Mean ±SE).
